# Orofacial injuries in pregnant women victims of domestic violence: a cross-sectional study at a forensic medicine service in Southern Brazil, 2015-2023

**DOI:** 10.1590/S2237-96222025v34e20240772.en

**Published:** 2025-10-27

**Authors:** Henrique Freitas Jalil, Leticia Regina Morello Sartori, Helena Bork Kohn, Eliza Corina Lopes Gomes, Cristina Braga Xavier

**Affiliations:** 1 Universidade Federal de Pelotas, Faculdade de Odontologia, Pelotas, RS, Brazil; 2 Universidade Federal de Pelotas, Programa de Pós-Graduação em Odontologia, Pelotas, RS, Brazil; 3 Universidade Católica de Pelotas, Faculdade de Medicina, Pelotas, RS, Brazil; 4 Instituto Geral de Perícias, Departamento de Perícias do Interior, Pelotas, RS, Brazil

**Keywords:** Domestic Violence, Violence Against Women, Pregnant People, Health Information Systems, Cross-Sectional Studies, Violencia Doméstica, Violencia contra la Mujer, Personas Embarazadas, Sistemas de Información en Salud, Estudios Transversales

## Abstract

**Objectives:**

To describe the prevalence and investigate whether demographic and circumstantial factors might be associated with the presence of orofacial injuries in cases of family violence against pregnant women examined at the Pelotas Forensic Medicine Service.

**Methods:**

This was a cross-sectional study, developed based on the analysis of expert witness examination reports in cases of family violence against pregnant women between 2015 and 2023. Presence of orofacial injuries was taken as the outcome variable. Predictor variables related to the demographic characteristics of the victims and the violent episodes described in the reports were collected. Descriptive and bivariate association analyses were conducted considering a 5% significance level.

**Results:**

A total of 103 reports were included in this study. Most of the reports were recorded between 2016 and 2018 (61.1%). Following expert witness examination, 77.7% of the investigations were conducted by the Police Station for Women. Regarding age, 29.1% of the victims were between 19 and 25 years old. The perpetrator was named in 30.1% of the reports. Orofacial injuries were found in 46.6% of the victims, being the second most frequent type after injuries to the upper limbs (59.2%). The upper third of the face was affected in 62.5% of the cases, with injuries predominantly to extra-oral soft tissues. No associations were found between the predictor variables analyzed and the presence of injuries.

**Conclusion:**

High prevalence of orofacial injuries was found among pregnant women victims of domestic violence examined at the Pelotas Forensic Medicine Service, with no associations found with the predictor variables analyzed.

## Resumen

**Objetivos:**

Describir la prevalencia e investigar si factores demográficos y circunstanciales se asociarían con la presencia de lesiones orofaciales en casos de violencia familiar contra embarazadas examinadas en el Servicio de Medicina Legal de Pelotas.

**Métodos:**

Estudio transversal, basado en el análisis de informes periciales en casos de violencia familiar contra embarazadas entre 2015 y 2023. La presencia de lesiones orofaciales se consideró la variable de resultado, y se recopilaron variables predictoras relacionadas con las características demográficas de la víctima y el episodio violento presentes en el informe. Se realizaron análisis de asociación descriptivos y bivariados, considerando un nivel de significancia del 5%.

**Resultados:**

Se incluyeron en este estudio un total de 103 informes. La mayoría de los informes se registraron entre 2016 y 2018 (61,1%). Después de la evaluación pericial, el 77,7% de las investigaciones fueron realizadas por la Comisaría de la Mujer. En cuanto a la edad, el 29,1% de las víctimas tenían entre 19 y 25 años. El agresor fue citado en el 30,1% de los informes. Se observaron lesiones orofaciales en el 46,6% de las víctimas, siendo el segundo tipo más frecuente después de las lesiones en los miembros superiores (59,2%). El tercio superior de la cara se vio afectado en el 62,5% de los casos, con lesiones predominantemente de tejidos blandos extraorales. No se observaron asociaciones entre las variables predictoras analizadas y la presencia de lesiones.

**Conclusión:**

Se observó una alta prevalencia de lesiones orofaciales entre las mujeres embarazadas víctimas de violencia doméstica que fueron examinadas en el Servicio de Medicina Legal de Pelotas, sin observarse asociaciones con las variables predictoras analizadas.

**Ethical aspects**

This research respected ethical principles, having obtained the following approval data:

Research Ethics Committee: Universidade Federal de Pelotas

Opinion number: 6,505,512

Approval date: 14/11/2023

Certificate of Submission for Ethical Appraisal: 50199621.7.0000.5318

Informed Consent Form: Exempted, the study used secondary data from services that make their medical records available for data collection and subsequent analysis.

## Introduction 

Violence against women is a human rights violation and a significant public health problem that affects one in three women globally and also in the Americas, in the form of physical violence, sexual violence, or both (1). Among the factors that increase vulnerability to violence, pregnancy can represent a period when violent episodes begin or escalate (2). Global prevalence of intimate partner violence during pregnancy has been estimated at 25.0% (3). In Brazil, some locations have presented high percentages: in Caxias, in the state of Maranhão, between 2019 and 2020, 24.1% of pregnant women reported psychological violence, 9.5% physical violence, and 3.0% sexual violence (4). In Cariacica, in the state of Espírito Santo, in 2017, the respective percentages were 16.1%, 7.6%, and 2.7% (5).

In addition to its high prevalence, maternal-fetal exposure to violence has repercussions on poorer mental health, physical injuries, reproductive complications, and maternal-fetal death (6,7). Regarding physical injuries, a multicenter study conducted with hospital data from California, United States, between 2016 and 2021, found that injuries to the head and abdomen were highly prevalent among pregnant women victims of intimate partner violence (8). However, that study did not address orofacial injuries (8). In data on hospital care (9) and expert witness examinations (10-13) related to women victims of domestic violence, information on oral and maxillofacial injuries is not specifically linked to the gestational period.

As such, this study aimed to describe the prevalence and investigate whether demographic and circumstantial factors are associated with the presence of orofacial injuries arising from cases of family violence against pregnant women examined at the Pelotas Forensic Medicine Service. 

## Methods 

In this study we followed “The Strengthening the Reporting of Observational Studies in Epidemiology (STROBE) statement (14).

### Study design and setting

This was a cross-sectional study based on digital expert witness examination reports from the Pelotas Forensic Medicine Service. This service is subordinated to the Rio Grande do Sul State General Institute of Expert Witness Examinations, responsible for expert examinations that serve to inform criminal prosecution proceedings (15).

### Study participants and size 

All expert witness examination reports recorded from January 2015 to December 2023 on the Pelotas Forensic Medicine Service system, referring to bodily injuries to pregnant women resulting from domestic violence, were considered eligible. In accordance with the service’s internal protocols, classification of reports as relating to this type of violence is done by a forensic physician, based on the victim’s verbal report on the existence of a family relationship with her aggressor (for example, partner, ex-partner or other family member), during her consultation with this physician.

### Data source and collection and bias

The data were collected from the internal system of the Pelotas Forensic Medicine Service by a trained researcher, with subsequent review by a researcher experienced in secondary data. This double check sought to minimize information biases.

### Variables and categories

The outcome variable was the presence of orofacial injuries (no, yes). The characteristics of the injuries were described based on their location in the upper third (forehead to zygoma), middle third (zygoma to upper lip) and lower third of the face (mandibular region) and based on the involvement of extra-oral soft tissues, intra-oral soft tissues, presence of bone fractures and traumatic dental injuries, all categorized dichotomously (no, yes).

The predictor variables included: the year of the report (categorized as: 2016-2018, 2019-2021, 2022-2023); the police station that requested the expert witness examination and the police station that conducted the investigation (categorized as: non-specialized police station, Police Station for Women, Police Station for Children and Adolescents); the place in which the expert witness examination requisition was made (Pelotas, other municipality); whether there was of discussion of the expert witness examination report, this being a field filled in by the forensic physician when additional examinations are necessary (no; yes, request for additional obstetric or fetal examination; yes, request for obstetric or fetal and other examination); the age of the victim (in years: 15-18, 19-25, 26-30, 31-40, 41 or over); the perpetrators being named in the report (no, yes); and the presence of injuries to the skull, neck, thorax, abdomen, upper limbs and lower limbs, all categorized dichotomously (no, yes).

### Statistical analysis

The STATA 18.0 statistical package (STATA Corp., College Station, Texas, United States), was used to obtain absolute and relative frequencies and 95% confidence intervals of the variables of interest. Bivariate associations were tested using Fisher’s exact test, considering a 5% significance level.

## Results 

Between 2015 and 2023, 110 expert witness examination reports were recorded as cases of domestic violence against pregnant women. Of these, six cases were excluded, given that the individuals examined were male (four in 2016, one in 2017 and one in 2019), and one case was excluded due to the incompatibility of the age of the person examined with the possibility of pregnancy. This resulted in 103 (93.6%) expert witness examination reports comprising the sample of this study.

A descriptive analysis of the variables related to the records is shown (Table 1). 61.1% of the expert witness examinations were performed between 2016 and 2018. 73.8% of the victims were referred by non-specialized police stations, however the investigations were predominantly conducted by the Police Station for Women (77.7%). 29.1% of the victims were between 19 and 25 years old. The perpetrators were named in 30.1% of the reports. No associations were found between these variables and the presence of orofacial injuries (Table 1).

**Table 1:** Absolute frequencies (n), relative frequencies (%) and 95% confidence intervals (95%CI) of the study variables and bivariate analysis with the presence of orofacial injuries. Pelotas, 2015-2023 (n=103)

Variable	Total		Orofacial injuries		p-value
	n (%)	95%CI	No n (%)	Yes n (%)	
Year recorded					0.546
2016-2018	63 (61.1)	51.0; 70.6	32 (50.8)	31 (49.2)	
2019-2021	22 (21.4)	13.9; 30.5	14 (63.6)	8 (36.4)	
2022-2023	18 (17.5)	10.6; 26.2	9 (50.0)	9 (50.0)	
Police station that requested the expert witness report					0.311
Non-specialized police station	76 (73.8)	64.2; 81.9	43 (56.6)	33 (43.4)	
Police Station for Women	26 (25.3)	17.2; 34.7	12 (46.1)	14 (53.8)	
Police Station for Children and Adolescents	1 (0.9)	0.2; 5.3	0 (0.0)	1 (100)	
Police station that conducted the investigation					0.581
Non-specialized police station	19 (18.4)	11.5; 27.3	10 (52.6)	9 (47.4)	
Police Station for Women	80 (77.7)	68.4; 85.3	44 (55.0)	36 (45.0)	
Police Station for Children and Adolescents	4 (3.9)	1.1; 9.6	1 (25.0)	3 (75.0)	
Place examination requisition made					0.414
Pelotas	97 (94.2)	87.7; 97.8	53 (54.6)	44 (45.4)	
Other municipality	6 (5.8)	2.2; 12.2	2 (33.3)	4 (66.7)	
Discussion of the report					0.873
No	91 (88.3)	80.5; 93.8	48 (52.7)	43 (47.2)	
Yes, requisition made for additional obstetric or fetal examination	10 (9.7)	4.7; 17.1	6 (60.0)	4 (40.0)	
Yes, requisition made for additional obstetric or fetal and other examination	2 (1.9)	0.2; 6.8	1 (50.0)	1 (50.0)	
Victims age (years)					0.786
15-18	19 (18.4)	11.5; 27.3	10 (52.6)	9 (47.4)	
19-25	30 (29.1)	20.6; 38.9	16 (53.3)	14 (46.7)	
26-30	23 (22.3)	14.7; 31.6	10 (43.5)	13 (56.5)	
31-40	22 (21.4)	13.9; 30.5	13 (59.1)	9 (40.9)	
41 or over	9 (8.7)	4.1; 15.9	6 (66.7)	3 (33.3)	
Perpetrators named in the report					0.833
No	72 (69.9)	60.1; 78.5	39 (54.2)	33 (45.8)	
Yes	31 (30.1)	21.4; 39.9	16 (51.6)	15 (48.4)	
					

Among the pregnant women examined, 46.6% presented orofacial injuries (Table 2). This was the second most frequent injury site, after the upper limbs (59.2%). The abdomen was the least affected region (6.8%). No associations were found between the presence of injuries in other body regions and orofacial regions. 

**Table 2:** Absolute frequencies (n), relative frequencies (%) and 95% confidence intervals (95%CI) of the body region in which injuries were recorded and bivariate analysis with the presence of orofacial injuries. Pelotas, 2015-2023 (n=103)

Variable	Total		Orofacial injuries		p-value
	n (%)	95%CI	No n (%)	Yes n (%)	
Skull					0.767
No	90 (87.4)	79.4; 93.1	49 (54.4)	41 (45.6)	
Yes	13 (12.6)	6.9; 20.6	6 (46.1)	7 (53.8)	
Neck					0.767
No	91 (88.3)	80.5; 93.8	48 (52.7)	43 (47.2)	
Yes	12 (11.7)	6.2; 19.5	7 (58.3)	5 (41.6)	
Thorax					0.249
No	89 (86.4)	78.2; 92.4	50 (56.2)	39 (43.8)	
Yes	14 (13.6)	7.6; 21.7	5 (35.7)	9 (64.3)	
Abdomen					1.000
No	96 (93.2)	86.5; 97.2	51 (53.1)	45 (46.9)	
Yes	7 (6.8)	2.8; 13.5	4 (57.1)	3 (42.9)	
Upper limbs					0.228
No	42 (40.8)	31.2; 50.9	19 (45.2)	23 (54.8)	
Yes	61 (59.2)	49.1; 68.8	36 (59.0)	25 (41.0)	
Lower limbs					0.204
No	70 (68.0)	58.0; 76.8	34 (48.6)	36 (51.4)	
Yes	33 (32.0)	23.2; 41.9	21 (63.6)	12 (36.4)	
Face			-	-	-
No	55 (53.4)	43.3; 63.3			
Yes	48 (46.6)	36.7; 56.7			
					

**Table 3:** Absolute frequencies (n), relative frequencies (%) and 95% confidence intervals (95%CI) of the orofacial injury characteristics. Pelotas, 2015-2023 (n=48)

Variable/category	n (%)	95%CI
Upper third of the face		
No	18 (37.5)	23.9; 52.6
Yes	30 (62.5)	47.3; 76.0
Middle third of the face		
No	33 (68.7)	53.7; 81.3
Yes	15 (31.3)	18.6; 4.2
Lower third of the face		
No	29 (60.4)	45.3; 74.2
Yes	19 (39.6)	25.8; 54.7
Extra-oral soft tissues		
No	4 (8.3)	2.3; 20.0
Yes	44 (91.7)	80.0; 97.7
Intra-oral soft tissues		
No	37 (77.1)	62.7; 88.0
Yes	11 (22.9)	12.0; 37.3
Traumatic dental injuries		
No	47 (97.9)	88.9; 99.9
Yes	1 (2.1)	0.1; 11.1

The characteristics of the reported orofacial injuries are shown in detail (Table 3). The upper third of the face was affected in 62.5% of cases, and extraoral soft tissues in 91.7% of cases. One case presented traumatic dental injury.

## Discussion 

This study identified high prevalence of orofacial injuries among the pregnant women victims of domestic violence examined, but no significant associations were observed with the predictor variables analyzed. Considering the demographic and circumstantial profile, most of the reports referred to young adult pregnant women, with expert witness examinations carried out between 2016 and 2018, whereby the perpetrator was not named for the most part and referrals were frequently made by non-specialized police stations, but investigations were mostly conducted by the Police Station for Women.

Expert witness examination reports are an important source of secondary data on the repercussions of physical violence, representing the category of cases of violence that are taken to court (13,15). The findings of this study provide relevant evidence about the prevalence and pattern of orofacial injuries in pregnant victims examined at a forensic medicine service in Southern Brazil. However, some limitations should be noted. Due to the characteristics of the records, ethnic-racial and socioeconomic information and unfavorable maternal-fetal health outcomes were not available for collection. Few of the records included in the study provided identification of the perpetrators, this being a result very similar to that found in a study carried out on examination reports of violence against women held at the São Luís Forensic Medicine Service in Northeast Brazil (10). In both studies, it was also not possible to directly establish the relationship between the perpetrator and the victim (10).

At the São Luís Forensic Medicine Service, it was identified that 26.0% of women victims of physical violence between 2010 and 2013 had suffered oral and maxillofacial injuries (10). In our study, prevalence of orofacial injuries among pregnant women was 46.6%. This higher prevalence rate found by us may reflect both a more recent period of data collection, 2015 to 2023, and potential particularities of physical violence against pregnant women that should be better investigated in future studies.

Higher proportions of extra-oral injuries were observed, mainly in the upper third of the face, which includes the orbital and forehead regions, which corroborates previous findings (10,11). The lower prevalence of reported intra-oral injuries and traumatic dental injuries may be a combined result of the characteristics of the data source – a Forensic Medicine Service that does not have a forensic dentistry department –, as well as the sample size of this study (12,13).

Although the descriptive pattern of the circumstances of the violent episodes recorded in the reports corroborates information already reported in the literature, such as the higher prevalence of violence against women in young adulthood (10-12), this study did not find associations with any of the predictors assessed. Potentially, the sample size of this study influenced this finding. The lack of association between orofacial injuries and those in other regions of the body may further suggest that the patterns of physical injuries in pregnant women vary, which implies the need for multidisciplinary health care to ensure adequate guidance and support (7). Most of the subsequent investigations were conducted by the Police Station for Women. This scenario reflects the importance of the specialized care network in situations of family violence through specific public policies (13).

To conclude, there was a 46.6% prevalence rate for orofacial injuries among pregnant women victims of domestic violence and examined at the Pelotas Forensic Medicine Service between 2015 and 2023. The upper third of the face and extra-oral soft tissues were the most affected. There was no association with the predictors assessed.
