# Supplementary material for: Orofacial injuries in pregnant women victims of domestic violence: a cross-sectional study at a forensic medicine service in Southern Brazil, 2015-2023
Source: Epidemiol Serv Saude. 2025 Oct 27;34:e20240772. doi: 10.1590/S2237-96222025v34e20240772.en (PMC12560224; doi:10.1590/S2237-96222025v34e20240772.en)
Supplement: Translation

# Translation: Lesões orofaciais em gestantes vítimas de violência familiar: estudo transversal em um posto médico legal no Sul do Brasil, 2015-2023

**DOI:** 10.1590/S2237-96222025v34e20240772.pt

**Authors:** Henrique Freitas Jalil, Leticia Regina Morello Sartori, Helena Bork Kohn, Eliza Corina Lopes Gomes, Cristina Braga Xavier

**Affiliations:** 1 Universidade Federal de Pelotas, Faculdade de Odontologia, Pelotas, RS, Brasil; 2 Universidade Federal de Pelotas, Programa de Pós-Graduação em Odontologia, Pelotas, RS, Brasil; 3 Universidade Católica de Pelotas, Faculdade de Medicina, Pelotas, RS, Brasil; 4 Instituto Geral de Perícias, Departamento de Perícias do Interior, Pelotas, RS, Brasil

**Keywords:** Violência Doméstica, Violência Contra a Mulher, Gestantes, Sistemas de Informações em Saúde, Estudos Transversais

## Abstract

**Objetivos:**

Descrever a prevalência e investigar se fatores demográficos e circunstanciais estariam associados à presença de lesões orofaciais em casos de violência familiar contra mulheres gestantes periciadas no Posto Médico Legal de Pelotas.

**Métodos:**

Tratou-se de estudo transversal, desenvolvido a partir da análise dos laudos das perícias em casos de violência familiar contra gestantes entre 2015 e 2023. A presença de lesões orofaciais foi considerada a variável de desfecho, e foram coletadas variáveis preditoras relacionadas às características demográficas da vítima e do episódio violento presentes no laudo. Foram conduzidas análises descritivas e de associação bivariada considerando o nível de significância de 5%.

**Resultados:**

Foram incluídos 103 laudos neste estudo. A maioria dos laudos foi registrada entre 2016 e 2018 (61,1%). Após a perícia, 77,7% dos inquéritos foram conduzidos pela Delegacia da Mulher. Em relação à idade, 29,1% das vítimas tinham entre 19 e 25 anos. O perpetrador foi citado em 30,1% dos laudos. Lesões orofaciais foram observadas em 46,6% das vítimas, sendo o segundo tipo mais frequente após as lesões nos membros superiores (59,2%). O terço superior da face foi afetado em 62,5% dos casos, em lesões predominantemente de tecidos moles extraorais. Não foram observadas associações entre as variáveis preditoras analisadas e a presença de lesões.

**Conclusão:**

Observou-se alta prevalência de lesões orofaciais entre gestantes vítimas de violência familiar periciadas no Posto Médico Legal de Pelotas, não sendo observadas associações com as variáveis preditoras analisadas.

**Aspectos éticos**

A presente pesquisa respeitou os princípios éticos, obtendo os seguintes dados de aprovação:

Comitê de ética em pesquisa: Universidade Federal de Pelotas

Número do parecer: 6.505.512

Data de aprovação: 14/11/2023

Certificado de apresentação de apreciação ética: 50199621.7.0000.5318

Registro do consentimento livre e esclarecido: Dispensado, o estudo utilizou dados secundários de serviços que disponibilizam os seus prontuários para a captação dos dados e posterior análise.

## Introdução 

A violência contra mulheres é uma violação aos direitos humanos e um problema relevante para a saúde pública que afeta, globalmente e nas Américas, uma em cada três mulheres sob a forma de violência física, sexual ou ambas (1). Entre os fatores que aumentam a vulnerabilidade à violência, a gestação pode representar um período de início ou intensificação dos episódios violentos (2). Foi estimada a prevalência global de violência por parceiro íntimo durante a gestação em 25,0% (3). No Brasil, algumas localidades apresentaram percentuais elevados: em Caxias, Maranhão, entre 2019 e 2020, 24,1% das gestantes relataram violência psicológica, 9,5% física e 3,0% sexual (4). Em Cariacica, Espírito Santo, em 2017, os percentuais foram 16,1%, 7,6% e 2,7% (5). 

Além das elevadas prevalências, a exposição materno-fetal à violência repercute em pior saúde mental, injúrias físicas, complicações reprodutivas e óbito materno-fetal (6,7). Considerando as injúrias físicas, estudo multicêntrico realizado com dados hospitalares da Califórnia, Estados Unidos, entre 2016 e 2021, observou que injúrias em cabeça e abdome tiveram alta prevalência entre gestantes vítimas de violência por parceiro íntimo (8). Contudo, este estudo não abordou lesões orofaciais (8). Em dados oriundos de atendimentos hospitalares (9) e periciais (10-13) relacionados a mulheres vítimas de violência familiar, as informações sobre lesões bucomaxilofaciais não são especificadas considerando-se o período gestacional.

Desta forma, este estudo objetivou descrever a prevalência e investigar se fatores demográficos e circunstanciais estão associados à presença de lesões orofaciais oriundas de casos de violência familiar contra gestantes periciadas no Posto Médico Legal de Pelotas. 

## Métodos 

O protocolo “The Strengthening the Reporting of Observational Studies in Epidemiology (STROBE) foi seguido neste estudo (14).

### Delineamento do estudo e contexto

Tratou-se de estudo transversal baseado em laudos periciais digitais do Posto Médico Legal de Pelotas. Esse órgão é vinculado ao Instituto-Geral de Perícias do Rio Grande do Sul, responsável por exames periciais que subsidiam processos criminais (15). 

### Participantes e tamanho do estudo 

Foram considerados elegíveis todos os laudos periciais registrados de janeiro de 2015 a dezembro de 2023 no sistema do Posto Médico Legal de Pelotas, referentes a lesões corporais em gestantes decorrentes de violência familiar. Nos protocolos internos, a classificação dos laudos nesse tipo de violência é realizada pelo médico perito, com base no relato verbal da vítima sobre a existência de vínculo familiar com o agressor (por exemplo, parceiro, ex-parceiro ou outro familiar), no atendimento pericial.

### Fonte, coleta dos dados e viés

A coleta foi realizada no sistema interno do Posto Médico Legal de Pelotas por um pesquisador treinado, com revisão posterior por pesquisadora experiente em dados secundários. Essa dupla checagem buscou minimizar vieses de informação.

### Variáveis e categorias

A variável de desfecho foi a presença de lesões orofaciais (não, sim). As características das lesões foram descritas a partir da localização em terço superior (região da fronte até o zigoma), médio (zigoma até lábio superior) e inferior da face (região mandibular) e a partir do acometimento de tecidos moles extraorais, tecidos moles intraorais, presença de fraturas ósseas e de lesões dentárias traumáticas, todas categorizadas de forma dicotômica (não, sim).

As variáveis preditoras incluíram: o ano do registro (categorizados em: 2016-2018, 2019-2021, 2022-2023); a delegacia que solicitou a perícia e a de condução do inquérito (categorizadas em: delegacia não especializada, Delegacia da Mulher, Delegacia da Criança e do Adolescente); o local de requisição (Pelotas, outro município); a presença de discussão do laudo, campo preenchido pelo perito quando são necessários exames adicionais (não; sim, requisição de complemento obstétrico ou fetal; sim, requisição de complemento obstétrico ou fetal e outro); a idade da vítima (em anos: 15-18, 19-25, 26-30, 31-40, 41 ou mais); a menção dos perpetradores no laudo pericial (não, sim); e a presença de lesões em região de crânio, pescoço, tórax, abdome, membros superiores e inferiores, todas categorizadas de forma dicotômica (não, sim).

### Análise estatística

No pacote estatístico STATA 18.0 (STATA Corp., College Station, Texas, Estados Unidos), frequências absolutas e relativas e intervalos de confiança de 95% das variáveis de interesse foram obtidos. Associações bivariadas foram testadas através do teste exato de Fisher, considerando nível de significância de 5%. 

## Resultados 

Entre 2015 e 2023, foram registrados 110 laudos de perícias médicas registradas como violência familiar contra gestantes. Destes, seis casos foram excluídos, visto que os periciados eram do sexo masculino (quatro em 2016, um em 2017 e um em 2019), e um caso foi excluído devido à incompatibilidade da idade da periciada com a possibilidade de gestação. Ao final, 103 (93,6%) laudos periciais compuseram a amostra deste estudo. 

Apresentou-se a análise descritiva das variáveis relacionadas ao registro (Tabela 1). Entre 2016 e 2018, ocorreram 61,1% das perícias. Foram encaminhadas por delegacias não especializadas 73,8% das vítimas, contudo os inquéritos foram predominantemente conduzidos pela Delegacia da Mulher (77,7%). Entre as vítimas, 29,1% tinham idade entre 19 e 25 anos. Houve menção dos perpetradores em 30,1% dos laudos. Não foram observadas associações dessas variáveis com a presença de lesões orofaciais (Tabela 1). 

**Tabela 1:** Frequências absolutas (n), frequências relativas (%) e intervalos de confiança de 95% (IC95%) das variáveis do estudo e análise bivariada com a presença de lesões orofaciais. Pelotas, 2015-2023 (n=103)

Variável	Total		Lesões orofaciais		p-valor
	n (%)	IC95%	Não n (%)	Sim n (%)	
Ano de registro					0,546
2016-2018	63 (61,1)	51,0; 70,6	32 (50,8)	31 (49,2)	
2019-2021	22 (21,4)	13,9; 30,5	14 (63,6)	8 (36,4)	
2022-2023	18 (17,5)	10,6; 26,2	9 (50,0)	9 (50,0)	
Delegacia que solicitou a perícia					0,311
Delegacia não especializada	76 (73,8)	64,2; 81,9	43 (56,6)	33 (43,4)	
Delegacia da Mulher	26 (25,3)	17,2; 34,7	12 (46,1)	14 (53,8)	
Delegacia da Criança e do Adolescente	1 (0,9)	0,2; 5,3	0 (0,0)	1 (100)	
Delegacia de condução do inquérito					0,581
Delegacia não especializada	19 (18,4)	11,5; 27,3	10 (52,6)	9 (47,4)	
Delegacia da Mulher	80 (77,7)	68,4; 85,3	44 (55,0)	36 (45,0)	
Delegacia da Criança e do Adolescente	4 (3,9)	1,1; 9,6	1 (25,0)	3 (75,0)	
Local de requisição					0,414
Pelotas	97 (94,2)	87,7; 97,8	53 (54,6)	44 (45,4)	
Outro município	6 (5,8)	2,2; 12,2	2 (33,3)	4 (66,7)	
Discussão do laudo					0,873
Não	91 (88,3)	80,5; 93,8	48 (52,7)	43 (47,2)	
Sim, requisição de complemento obstétrico ou fetal	10 (9,7)	4,7; 17,1	6 (60,0)	4 (40,0)	
Sim, requisição de complemento obstétrico ou fetal e outro	2 (1,9)	0,2; 6,8	1 (50,0)	1 (50,0)	
Idade da vítima (anos)					0,786
15-18	19 (18,4)	11,5; 27,3	10 (52,6)	9 (47,4)	
19-25	30 (29,1)	20,6; 38,9	16 (53,3)	14 (46,7)	
26-30	23 (22,3)	14,7; 31,6	10 (43,5)	13 (56,5)	
31-40	22 (21,4)	13,9; 30,5	13 (59,1)	9 (40,9)	
41 ou mais	9 (8,7)	4,1; 15,9	6 (66,7)	3 (33,3)	
Perpetradores citados no laudo					0,833
Não	72 (69,9)	60,1; 78,5	39 (54,2)	33 (45,8)	
Sim	31 (30,1)	21,4; 39,9	16 (51,6)	15 (48,4)	

Entre as gestantes periciadas, 46,6% apresentaram lesões orofaciais (Tabela 2). Essa foi a segunda localização mais frequente, atrás apenas dos membros superiores (59,2%). O abdome foi a região menos afetada (<6,8%). Não foram observadas associações entre a presença de lesões em outras regiões corporais e orofaciais. 

**Tabela 2:** Frequências absolutas (n), frequências relativas (%) e intervalos de confiança de 95% (IC95%) da região corporal em que lesões foram registradas e análise bivariada com a presença de lesões orofaciais. Pelotas, 2015-2023 (n=103)

Variável	Total		Lesões orofaciais		p-valor
	n (%)	IC95%	Não n (%)	Sim n (%)	
Crânio					0,767
Não	90 (87,4)	79,4; 93,1	49 (54,4)	41 (45,6)	
Sim	13 (12,6)	6,9; 20,6	6 (46,1)	7 (53,8)	
Pescoço					0,767
Não	91 (88,3)	80,5; 93,8	48 (52,7)	43 (47,2)	
Sim	12 (11,7)	6,2; 19,5	7 (58,3)	5 (41,6)	
Tórax					0,249
Não	89 (86,4)	78,2; 92,4	50 (56,2)	39 (43,8)	
Sim	14 (13,6)	7,6; 21,7	5 (35,7)	9 (64,3)	
Abdome					1,000
Não	96 (93,2)	86,5; 97,2	51 (53,1)	45 (46,9)	
Sim	7 (6,8)	2,8; 13,5	4 (57,1)	3 (42,9)	
Membros superiores					0,228
Não	42 (40,8)	31,2; 50,9	19 (45,2)	23 (54,8)	
Sim	61 (59,2)	49,1; 68,8	36 (59,0)	25 (41,0)	
Membros inferiores					0,204
Não	70 (68,0)	58,0; 76,8	34 (48,6)	36 (51,4)	
Sim	33 (32,0)	23,2; 41,9	21 (63,6)	12 (36,4)	
Face			-	-	-
Não	55 (53,4)	43,3; 63,3			
Sim	48 (46,6)	36,7; 56,7			

Foram detalhadas as características das lesões orofaciais reportadas (Tabela 3). O terço superior foi acometido em 62,5% dos casos, e tecidos moles extraorais, em 91,7%. Um caso apresentou lesão dentária traumática. 

**Tabela 3:** Frequências absolutas (n), frequências relativas (%) e intervalos de confiança de 95% (IC95%) das características das lesões orofaciais. Pelotas, 2015-2023 (n=48)

Variável/categoria	n (%)	IC95%
Terço superior da face		
Não	18 (37,5)	23,9; 52,6
Sim	30 (62,5)	47,3; 76,0
Terço médio da face		
Não	33 (68,7)	53,7; 81,3
Sim	15 (31,3)	18,6; 4,2
Terço inferior da face		
Não	29 (60,4)	45,3; 74,2
Sim	19 (39,6)	25,8; 54,7
Tecidos moles extraorais		
Não	4 (8,3)	2,3; 20,0
Sim	44 (91,7)	80,0; 97,7
Tecidos moles intraorais		
Não	37 (77,1)	62,7; 88,0
Sim	11 (22,9)	12,0; 37,3
Lesões dentárias traumáticas		
Não	47 (97,9)	88,9; 99,9
Sim	1 (2,1)	0,1; 11,1
		

## Discussão 

Este estudo identificou alta prevalência de lesões orofaciais entre gestantes vítimas de violência familiar periciadas, sem, contudo, serem observadas associações significativas com as variáveis preditoras analisadas. Considerando o perfil demográfico e circunstancial, grande parte dos laudos se referiu a gestantes adultas jovens, com perícias realizadas entre 2016 e 2018, com perpetrador não mencionado e com encaminhamentos frequentemente realizados por delegacias não especializadas, mas majoritariamente conduzidos pela Delegacia da Mulher.

Laudos periciais configuram fonte importante de dados secundários sobre as repercussões da violência física, representando a camada de casos de violência que adentram ao serviço judiciário (13,15). Os achados deste estudo forneceram evidências relevantes acerca da prevalência e do padrão de lesões orofaciais em vítimas gestantes periciadas em um posto médico legal no Sul do Brasil. Entretanto, algumas limitações devem ser sinalizadas. Em função das características do registro, informações étnico-raciais e socioeconômicas e desfechos desfavoráveis em saúde materno-fetal não estavam disponíveis para coleta. Poucos registros incluídos forneciam a identificação dos perpetradores, resultado muito similar ao encontrado em estudo realizado com laudos de violência contra a mulher do Instituto Médico Legal de São Luís (10); em ambos os estudos, também não foi possível estabelecer diretamente o vínculo do perpetrador com a vítima (10).

No Instituto Médico Legal de São Luís, foi identificado que 26,0% das mulheres vítimas de violência física entre 2010 e 2013 haviam sofrido lesões bucomaxilofaciais (10). Neste estudo, a prevalência de lesões orofaciais entre gestantes foi 46,6%. A maior prevalência observada pode refletir tanto um período mais recente de coleta de dados, 2015 a 2023, quanto potenciais particularidades da violência física contra gestantes que devem ser melhor investigadas em estudos futuros. 

Foram observadas maiores proporções de lesões extraorais, majoritariamente em terço superior da face que compreende as regiões orbital e frontal, o que corrobora achados prévios (10,11). A menor prevalência de reporte de lesões intraorais e de lesões dentárias traumáticas pode ser resultado combinado da característica da fonte dos dados – um Posto Médico Legal que não tem o setor de odontologia forense e do tamanho amostral deste estudo (12,13). 

Apesar de o padrão descritivo das circunstâncias do episódio violento registradas nos laudos corroborarem informações já apontadas na literatura, como a maior prevalência de violência contra mulheres em idade adulta jovem (10-12), este estudo não observou associações com nenhum dos preditores avaliados. Potencialmente, o tamanho amostral deste estudo influenciou esse achado. A falta de associação entre as lesões orofaciais e aquelas em outras regiões do corpo pode sugerir adicionalmente que os padrões de lesões físicas em gestantes variam, o que implica a necessidade de atenção multidisciplinar em saúde para garantir orientação e acolhimento adequados (7). Houve seguimento massivo das investigações através da Delegacia da Mulher. Esse cenário reflete a importância da rede de atendimento especializado em situações de violência familiar através de políticas públicas específicas (13). 

Em conclusão, houve prevalência de 46,6% de lesões orofaciais entre gestantes vítimas de violência familiar examinadas no Posto Médico Legal de Pelotas entre 2015 e 2023. O terço superior da face e tecidos moles extraorais foram os mais acometidos. Não houve associação com os preditores avaliados.
